# Genetic Architecture of Hybrid Male Sterility in *Drosophila*: Analysis of Intraspecies Variation for Interspecies Isolation

**DOI:** 10.1371/journal.pone.0003076

**Published:** 2008-08-27

**Authors:** Laura K. Reed, Brooke A. LaFlamme, Therese A. Markow

**Affiliations:** 1 Department of Ecology and Evolutionary Biology, University of Arizona, Tucson, Arizona, United States of America; 2 Department of Genetics, North Carolina State University, Raleigh, North Carolina, United States of America; 3 Department of Molecular Biology and Genetics, Cornell University, Ithaca, New York, United States of America; 4 Division of Biological Sciences, University of California San Diego, La Jolla, California, United States of America; University of Texas Arlington, United States of America

## Abstract

**Background:**

The genetic basis of postzygotic isolation is a central puzzle in evolutionary biology. Evolutionary forces causing hybrid sterility or inviability act on the responsible genes while they still are polymorphic, thus we have to study these traits as they arise, before isolation is complete.

**Methodology/Principal Findings:**

Isofemale strains of *D. mojavensis* vary significantly in their production of sterile F_1_ sons when females are crossed to *D. arizonae* males. We took advantage of the intraspecific polymorphism, in a novel design, to perform quantitative trait locus (QTL) mapping analyses directly on F_1_ hybrid male sterility itself. We found that the genetic architecture of the polymorphism for hybrid male sterility (HMS) in the F_1_ is complex, involving multiple QTL, epistasis, and cytoplasmic effects.

**Conclusions/Significance:**

The role of extensive intraspecific polymorphism, multiple QTL, and epistatic interactions in HMS in this young species pair shows that HMS is arising as a complex trait in this system. Directional selection alone would be unlikely to maintain polymorphism at multiple loci, thus we hypothesize that directional selection is unlikely to be the only evolutionary force influencing postzygotic isolation.

## Introduction

During the modern synthesis, Dobzhansky and Mayr proposed the Biological Species Concept that defines species by their ability to exchange genetic material within their group while being prevented from exchanging genetic material between groups [1], [2]. Reproductive isolating mechanisms subsequently have been the focus of extensive research such that a great deal now is understood about mechanisms of both pre and postzygotic isolation. Capturing the process of speciation early enough to determine the initial genetic causes of reproductive isolation, however, is challenging. Consequently a gap still remains in our understanding of the mechanisms underlying the very first stages of postzygotic isolation.

Postzygotic isolation exists when a hybrid, or its progeny, experience a reduction in fitness relative to the parental types. Whether the genetic variation needed for postzygotic isolation is segregating within species in the form of epistatic variation [3], [4] or instead arises as de novo mutations in allopatric populations [5] remains controversial. Thus, there is need for further empirical study to determine the roles of segregating and de novo polymorphism in speciation, and the underlying architecture of such variation [3].

Three general patterns of postzygotic isolation are well supported. First, Haldane's rule [6] states that decreases in hybrid fitness are more common in the heterogametic sex; second, hybrid sterility often appears earlier in species divergence than hybrid inviability [7], [8]; and third, heterogametic isolation generally appears earlier in speciation than homogametic isolation [8], [9]. Taken together, these patterns suggest that the earliest manifestation of postzygotic isolation likely will be hybrid sterility in the heterogametic sex. These patterns hold across diverse taxa in which the male is the heterogametic sex, such as mammals and *Drosophila* and interestingly, also for systems where the female is the heterogametic sex, such as birds and *Lepidoptera* [10]. Various theories exist about the basis for Haldane's rule [11]–[13]. The explanations with the most consistent empirical support revolve around the X (Z)-chromosome [14], leading to the expectation that the X-chromosome plays a substantial genetic role in the occurrence of postzygotic isolation in any given system.

The problem of how a trait that decreases the fitness of hybrids could evolve between two populations sharing a common ancestral genome without either population passing through a state of reduced fitness is solved by the Dobzhansky-Muller (DM) model of reproductive isolation [1], [15]–[17]. For example, if derived alleles of two loci arise independently in two populations then, upon meeting in a hybrid, they may be incompatible, leading to decreased fitness [18]. Several known interacting genomic regions have been implicated in hybrid incompatibilities [19]–[23]. In addition, the incompatible alleles of the *Lhr* and *Hmr* genes in the *Drosophila melanogaster – D. simulans* pair have been demonstrated functionally to cause hybrid male lethality [24].

Expanding the Dobzhansky-Muller model, Orr [18] proposed that the number of two locus incompatibilities between two populations will increase as a function of the square of their divergence times. If more complex genetic scenarios are considered, the accumulation of incompatibilities is even faster. Those genetic incompatibilities that initially defined the two species therefore, can rapidly become obscured by subsequent genetic differentiation. Efforts to elucidate the origin of isolation can be facilitated by identifying the architecture of postzygotic isolating mechanisms in incipient species, where the number of postzygotic incompatibilities are still few and, ideally, not yet fixed within the populations

A major lingering question in speciation genetics concerns the actual genetic architecture of postzygotic isolation as it first emerges. Does it have a simple or a complex genetic basis at the earliest stages of the speciation process? A burdensome disadvantage to genetic studies of postzygotic isolation is that the phenotype of interest, sterility or inviability, precludes direct genetic crossing schemes. Many successful studies of species pairs, in which the factors underlying isolation are effectively fixed, utilized the common occurrence of asymmetry [25] and/or Haldane's Rule. While the genetic dissections of incompatibility phenotypes by backcrosses and introgression [10], [21], [24], [26], [27] have contributed significantly to our current understanding of the genetics of postzygotic isolation, it is not without limitations. Since the crossing schemes employed potentially disrupted the very co-adapted gene complexes that might be critical to the F_1_ phenotype we cannot be certain that the genetic architecture for the incompatibility observed in the F_1_ hybrid has been fully characterized. Evidence of substantial within species epistasis contributing to postzygotic isolation has been demonstrated in *Tribolium* by joint-scaling analysis [28], [29] showing that co-adapted gene complexes may indeed be important in the architecture of hybrid incompatibilities.

In the present study, we circumvented the limitations of the hybrid phenotype by exploiting within-species polymorphism for between-species postzygotic isolation to determine the genetic architecture of segregating variation for F_1_ hybrid male sterility directly. We used *Drosophila mojavensis* and *D. arizonae*, a recently diverged species pair (0.66–1.2 my [30], [31]) and a model system for the study of speciation [32]. Crosses between *D. mojavensis* females and *D. arizonae* males exhibit widespread between-population [33], [34] and within-population variation for the HMS phenotype suggesting polymorphism at multiple loci within *D. mojavensis* [33]. The polymorphism for HMS shows that this trait is not yet fixed in *D. mojavensis*. Since intraspecific evolution is the process by which gene frequencies change, only a trait exhibiting genetic variation is capable of evolving. Thus HMS in this system is still capable of experiencing the forces of evolution (selection and/or drift) and can give insight into how HMS evolves. In a novel design, we have utilized the within-population variation for production of F_1_ HMS to determine the genomic regions and epistatic interactions that shape the architecture of postzygotic isolation within the F_1_ hybrid males. Below we address the following questions: What is the nature and number of polymorphic loci within *D. mojavensis* contributing to the HMS phenotype? And what can that tell us, in conjunction with other information about this species pair, about the evolution of HMS?

## Materials and Methods

### Lines used for mapping

Genetic lines were derived from lines used in the HMS polymorphism study by Reed and Markow [33]. Isofemale *D. mojavensis* lines were collected from Santa Catalina Island (CI) in April 2001. The Low (CI-10) line exhibiting low levels of HMS was inbred through full-sib mating for six generations and the High (CI-12) line exhibiting high levels of HMS was inbred for eight generations. The lines had minimum inbreeding coefficients of 0.734 and 0.826 respectively [35]. They showed significant differences from each other for HMS (presence of motile sperm) observed when females from those lines were crossed to *D. arizonae* males from an inbred line (minimum inbreeding coefficient of 0.859) collected at Peralta Canyon Trail Head, east of Phoenix, Arizona (AZ), in April 1997. All lines show normal male fertility when the males are genetically pure *D. mojavensis* or *D. arizonae* [33]. The sterility phenotype is only manifested in the species hybrid state. Homozygosity of the High line, used in the *D. mojavensis* genome sequencing project, had been confirmed by sequencing five marker loci in 10 females (L. Matzkin, *unpublished*) and an additional seven loci in 10 individuals by Agencourt Biosciences, and none showed sequence variation. The two *D. mojavensis* QTL lines were homokaryotypic, lacking inversion polymorphism. The lines used showed extreme contrasting HMS (sperm motility) phenotypes in heterospecific crosses (Table 1) as well as strong viability.

**Table 1:** Hybrid Sperm Motility in sons of mothers from the High and Low *D. mojavensis* lines and in the intercross populations of the High and Low lines, when females crossed to *D. arizonae* fathers.

	Proportion (N)		
Cross^1^	No Motility	Motility^2^	Standard Error
Low	0.54 (35)	0.46 (30)	0.062
F_1_	0.07 (11)	0.93 (154)	0.019
F_2_	0.15 (62)	0.85 (353)	0.017
RF_1_	0.07 (13)	0.93 (177)	0.018
RF_2_	0.13 (75)	0.87 (483)	0.014
High	0.09 (13)	0.91 (131)	0.024

1 Cross is Genotype of the *D. mojavensis* mother.

2 Motility is defined as a male having one or more motile sperm.

### Identifying Markers and Genotyping

We designed fluorescently-tagged PCR primers for flanking sequences of microsatellite loci identified by Ross *et al*. [36]. Additional candidate microsatellite sequences were drawn from Staten *et al.* [37]. Potential loci were genotyped in a panel of four individuals from each *D. mojavensis* line to confirm that the lines contained different alleles for the locus. We genotyped 132 potential loci but only found 25 that distinguished the High and Low lines (primer sequences not already described in Staten *et al*. [37] shown in Table S1). Polymorphic marker discovery was inhibited by the general deficit of neutral microsatellite variation in the wild population from which the mapping lines were derived [38]. We had markers on the X-chromosome and the four major autosomes but failed to find a marker on the 6^th^ dot chromosome. Markers were assigned to chromosome using one or more of the following methods. 1) BLASTing [39] the flanking sequence against the *D. melanogaster* genome sequence to identify which Muller element and thereby to what chromosome in *D. mojavensis* it belonged; 2) linkage mapping to allozyme markers assigned to chromosome by Zouros [40], [41]; 3) looking for close linkage between assigned markers and unassigned makers. Informative loci were multiplexed and PCR amplified using HotStarTaq DNA Polymerase (Qiagen Inc.). Genotyping was performed by the Genomic Analysis and Technology Core (GATC) at the University of Arizona using an ABI 3730 DNA Analyzer. Genotypes were read using Genotyper ver. 1.1 from Applied Biosystems.

### Crossing Design

**Figure 1:**
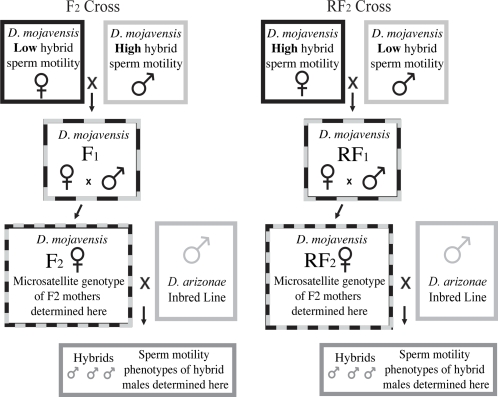
Crossing scheme. Design used to produce the recombinant *D. mojavensis* females to be mated to inbred *D. arizonae* males.

The crossing design used was an innovative modification of a standard F_2_ line cross (Figure 1). Five virgin F_2_ females, at six days post-eclosion, were paired with five sexually mature virgin male *D. arizonae* (AZ) for 48 hours. After 48 hours, the females were placed individually into vials, allowed to oviposit for four days, then removed and frozen for later genotyping. Virgin hybrid males were collected from those vials producing viable adults. Hybrid males were allowed to reach sexual maturity at 9–12 days post-eclosion before phenotyping. Use of the F_2_ mothers' genotypes allowed for a largest possible sample of recombinant genotypes (given limited resources for genotyping) while the use of the sons' phenotypes allowed for a good estimate of their mother's average phenotype, maximizing the quality and quantity of the data. A reciprocal design was also performed; instead of using Low females crossed to High males at the first step (F_2_ cross) High females were crossed to Low males (RF_2_ cross). A large number of attempted matings between F_2_ and RF_2_ females and *D. arizonae* males failed to produce offspring as was expected considering the significant prezygotic isolation between the species. Seven percent of the F_2_ (224 of 3214) and the 12% of the RF_2_ (278 of 2339) mothers produced larvae after being mated to *D. arizonae* males.

Mating and/or oviposition took place on standard opuntia-banana food (http://flyfood.arl.arizona.edu/opuntia.php3#s2.1) while virgins were maintained on standard cornmeal food (http://flyfood.arl.arizona.edu/cornmeal.php3). Cultures were maintained at 24+/−1 degree Celsius and under a strict 12h: 12h light: dark cycle.

### Phenotyping

Male *Drosophila* store mature sperm in a seminal vesicle, a structure at the base of each testis. To assess sperm motility, males were anesthetized with ether just prior to dissection. Testes and seminal vesicles were dissected from the male in sperm buffer (0.05 M Tris, 1.1% NaCL, 0.1% Glucose, 0.01% L-arginine, 0.01% L-lysine, pH 8.7). Reproductive organs were transferred to a fresh slide with an 11 microliter droplet of sperm buffer, stretched with forceps to be linear, and then placed under a coverslip. Pressing the coverslip down on the sample forced all sperm out of the seminal vesicle, allowing motility of the sperm to be observed under dark-field microscopy. The entire area where potentially motile sperm could be found was visually scanned and each seminal vesicle assigned an integer score from 0 to 6 on a sperm motility scale.

Unlike mammalian systems that have short sperm, *Drosophila* sperm are extremely long, forming a tangled wiggling knot, in which the movement of any individual sperm cannot be tracked. The zero-to-six scale is a reasonable means to approximate the amount of motility exhibited by *Drosophila* sperm, and is a refinement of the presence/absence of motility scheme used in many previous studies of sperm motility in *Drosophila* [33], [42], [43]. A quantitative measurement of sperm motility more suitable for quantitative genetic analysis results from the zero-to-six scale. A score of “0” meant there was no motility observed, “1” was one or two motile sperm, “2” being several motile sperm, “3” being several motile sperm in two or more areas of the field, “4” is three to four areas of moderate motility, “5” being several large areas of high motility, and “6” being wild-type where most of the field showed high levels of sperm motility (as is observed in non-hybrid males of these species). A male's phenotype was the average score of his two seminal vesicles. We phenotyped up to ten sons for each mother (mean family size of 3.4) and found no trends of decreasing motility scores with time (first verses second testis scored). The genotype of the male being scored was not known during phenotyping. A subset of samples were also scored and rescored in a randomized order while blind to sample identity. Replicate scores were highly correlated (R^2^ = 0.79, p<0.0001), validating this method for estimating *Drosophila* sperm motility suggesting its utility for future studies. Sperm motility is one phenotypic component of sterility. We have shown in previous work that motile sperm are required though not always sufficient for males to be able to reproduce in this system due to additional genetic polymorphism for other phenotypic components of sterility [33]. Studies of other phenotypic components of sterility would be interesting though not required to assess the importance of natural genetic variation for HMS as estimated by sperm motility in this system.

### Linkage Mapping

F_2_ or RF_2_ females that successfully produced hybrid male offspring were genotyped for the 25 microsatellite markers described above. Maximum likelihood marker (Figure S1) order was calculated for each chromosome and the Haldane mapping function was used to assign linkage distances between markers in centiMorgans (cM) using Mapmaker 3.0 [44].

Autosomal genotypes were pooled from the F_2_ and RF_2_ crosses for linkage map calculation while they are separated for the X-chromosome due to differences in X-chromosome inheritance patterns in the two crosses. Marker density was an average of 27.6 cM.

### QTL Mapping

QTL mapping was conducted using Windows QTL Cartographer 2.5 [45], [46]. We conducted composite interval mapping [47], [48] on the F_2_, RF_2_, and combined data sets using a common linkage maps for the autosomes and their respective linkage maps for the X-chromosome. Composite interval mapping (CIM) uses cofactors from other regions to control for genome wide influences when regression analysis at any given genomic location is being tested [47], [48]. For our study a multiple regression analysis at each 2 cM segment of each chromosome was performed using five randomly selected markers from elsewhere in the genome as cofactors to control for genetic effects in other genomic regions (Model 6 in QTLCartographer). A 10 cM region around the test location was excluded for selection of the five control markers. A likelihood ratio (LR) test was performed for each position comparing the null hypothesis that no QTL is present at that position to the alternative that there is a QTL present. To control for any violations of normality, multiple testing, or the possible disruption of the asymptotic distribution of the chi-squared test statistic by idiosyncrasies of the study (due to factors such as multiple QTL per chromosome or distorted segregation ratios), QTL peak significance was assessed by 1000 permutations [49].

Two datasets were used in mapping. In the one called *sons*, sons of each mother were analyzed with their mother's genotype and their individual motility phenotype to account for the within mother variation in her sons' motility scores. Analyses were also conducted on the second dataset using the mother's Best Linear Unbiased Prediction (BLUP) [50], [51], which considers both the mean and variance in offspring scores, as a summary of her “breeding quality” for hybrid male sperm motility calculated in the SAS/STAT Mixed Procedure (SAS 9.1.3, SAS Institute Inc. Cary, NC 2000–2004). The BLUP data is well suited to determine the basis of additive (heritable) variation while the *sons* data set is better for detecting genetic loci experiencing non-additive effects.

### Epistatic Interactions

We assessed epistatic interactions using methods modified from Moehring and Mackay [52] and Morgan and Mackay [53]. We tested for interaction effects between all pairs of markers using a repeated measures model of the form:
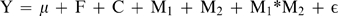
where F is the effect of mother, C is the effect of the direction of the cross (F_2_ vs. RF_2_), M_1_ is the effect of genotype at marker 1 and M_2_ is the effect of genotype at marker 2. All 300 possible two-way interactions were tested and significance of the interaction term (M_1_*M_2_) was assessed by Bonferroni correction and False Discovery Rate (FDR). All calculations were implemented using the SAS Mixed procedure (SAS 9.1.3, SAS Institute Inc., Cary, NC 2000–2004).

### Maternal Effects

Maternal effects were identified as a significant cross by main effect interaction. Hybrid male phenotype (n = 304) was regressed against the interaction between marker genotypes as the four main effect loci and the direction of the cross (F_2_ vs. RF_2_) in the multiple regression model of the form:
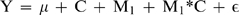
Where C in the direction of the cross and M_1_ is the genotype at the marker associated with one of the main effect QTL.

## Results

Inbred *D. mojavensis* lines showing High and Low phenotypic levels of HMS when crossed to *D. arizonae* were used in a standard F_2_ (and reciprocal) intercross QTL mapping design (Figure 1). Sperm motility in the parental, F_1_, RF_1_, F_2_, and RF_2_ lines are presented in Table 1. High and Low lines showed significant differences in the proportion of inter-species hybrid sons with motile sperm (91% and 46% respectively), and motility clearly was dominant in all hybrids (Table 1). Sperm motility is necessary but not always sufficient for fertility [33], thus motility is used as a correlate to absolute fertility. Sperm motility scores were normally distributed (Shapiro-Wilks W = 0.99, p = 0.38), with a mean of 2.35+/−0.08.

Female F_2_s (149 F_2_ and 155 RF_2_), of the species *D. mojavensis*, were crossed to male *D. arizonae* for QTL analysis. Sperm motility was scored, from 0 to 6, in the interspecific hybrid males. 304 F_2_ mothers were genotyped at 25 microsatellite markers across the genome and used to generate the linkage map. Phenotypes were mapped relative to genotype in two ways. One was to use each son's motility score, hereafter referred to as *sons*, as a sample of the mother's phenotype and the other was to map a mother's Best Linear Unbiased Prediction (BLUP) for her sons' motility scores. *Sons* better captures non-additive effects, while BLUP highlights the additive effects in the architecture.

### Main effects

**Figure 2:**
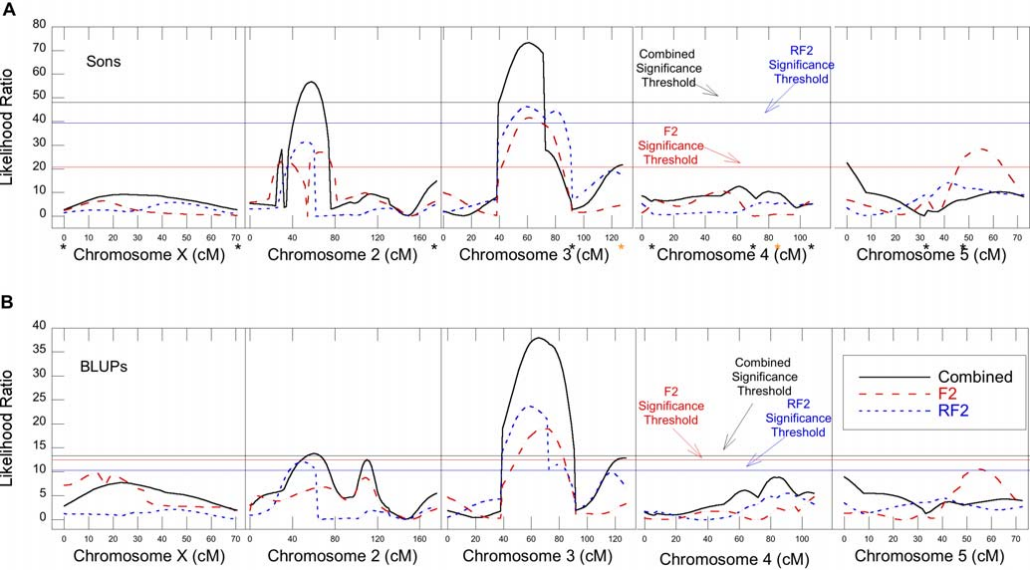
Composite interval mapping results for intraspecific variation for interspecific hybrid male sterility. Likelihood ratio values for the F_2_ (dashed red), RF_2_ (dashed blue), and combined (solid black) data sets are given for the X through 5^th^ chromosome. The X-chromosome scale is normalized to “combined” map length for the F_2_ and RF_2_ maps. The threshold for significance is the horizontal line (red- F_2_, blue- RF_2_, and black- combined). (a) Mapping results considering each son as measure of the mother's phenotype. Black asterisks indicate the location of markers with significant pairwise epistatic effects and yellow asterisks indicate two additional markers showing a significant deviation from the additive expectation. There is clear evidence for a QTL of major effect on the 3^rd^ chromosome for all data sets, on the 2^nd^ chromosome for the F_2_ and combined data sets, and on the 5^th^ for the F_2_ dataset. (b) Mapping results of the BLUP values for each mother. There is evidence for a QTL on major effect on the 3^rd^ chromosome in all three datasets and on the 2^nd^ chromosome in the combined dataset.

There were two striking outcomes of the main effects analysis; first that the F_2_ and RF_2_ mapping populations did not match more closely, and second, no main effects were detected on the X-chromosome. Main effect QTL were detected, however, on the 2^nd^, 3^rd^, and 5^th^ chromosomes (Table 2, Figure 2). Note that estimated effect sizes (percent of phenotypic variation explained) of all detected QTL are likely to be overestimates due to the Beavis effect [54], [55], thus, there are likely to be additional genetic factors contributing to observed differences between lines that were not detected in this study. Also the actual genotype of the hybrid males at a given marker can only fall into two possible genotypic classes, High or Low for the *D. mojavensis* half of their genome: they will be monomorphic with respect to the *D. arizonae* half of their genome. Thus, the variance due to non-additive effects identified in the QTL mapping corresponds to intra-genome epistasis rather then intra-locus dominance. Finally, remember that these two lines are only a sample from a larger population. There may be additional polymorphic factors on the X-chromosome or autosomes segregating in the population as a whole that were not captured by this biparental cross. One QTL with estimated effect size of 10.9–22.1% occurred on the 3^rd^ chromosome at about 60 cM and was observed in both datasets (*sons* and BLUP) and across all population samples (F_2_, RF_2_, and combined). The RF_2_ population sample did not reveal any other significant QTL.

**Table 2:** Maximum Likelihood Position and Effect Size for individual *son* and BLUP QTL found in Composite Interval Mapping.

Data Type^1^	Cross Type	Chromosome	Position (cM^2^)	LR^3^	Additive Effect	Non-Additive Effect	R^2^
IS	F_2_	2	32	23.68	0.24	−2.165	0.488
IS	F_2_	2	66	27.12	−0.381	2.164	0.506
IS	F_2_	3	61.2	41.64	−0.902	−0.137	0.157
IS	F_2_	5	54.5	28.39	−0.802	−0.427	0.096
IS	RF_2_	3	59.2	46.24	−0.79	0.357	0.127
IS	Combined	2	58	56.82	−0.392	2.225	0.553
IS	Combined	3	61.2	73.24	−0.739	0.132	0.109
BLUP	F_2_	3	69.2	18.98	−0.344	−0.046	0.122
BLUP	RF_2_	2	50	12.07	−0.255	1.101	0.593
BLUP	RF_2_	3	59.2	23.64	−0.476	0.294	0.221
BLUP	Combined	2	60	13.8	−0.197	0.921	0.443
BLUP	Combined	3	65.2	37.95	−0.406	0.138	0.153

1 Individual Son (IS) or the Best Linear Unbiased Prediction (BLUP) values.

2 cM is centiMorgans.

3 LR is likelihood ratio.

QTL were also identified on the 2^nd^ chromosome. Analysis of the *sons* dataset in the F_2_ and combined populations found two and one QTL on the 2^nd^ chromosome respectively (Table 2, Figure 2). The combined population *sons* QTL at 58 cM has a large non-additive effect of 2.23 motility units and estimated effect size is 55.3%. The two QTL found in the F_2_ population *sons* dataset (at 32 and 66 cM) also had large (and opposite) non-additive effects of −2.17 and 2.16 motility units, respectively. The RF_2_ *sons* dataset had an elevated likelihood ratio on the 2^nd^ chromosome but it did not reach significance. The BLUP datasets exposed a significant QTL on 2nd chromosome for the combined population (60 cM) and the RF_2_ population (50 cM) with a non-additive effect of 0.92 to 1.10 motility units, with estimated effect size of 44.3 to 59.3%. The lack of a significant 2^nd^ chromosome QTL in the F_2_ BLUP dataset can be explained by the apparent equal and opposite non-additive effects of the two QTL found in the *sons* dataset.

Chromosome five also contained a QTL, but only in the F_2_ population *sons* dataset. It was at 54.5 cM with a relatively large additive effect of −0.80 and moderate non-additive effect of −0.43 motility units with an estimated effect size of only 9%.

We tested for biases due to variation in family size by rerunning all analyses of both the BLUP and *sons* datasets on families limited to three randomly selected sons. All QTLs replicated with controlled family size (data not shown) so we are confident that the mapping results are not due to biases in family size. We also looked for any biases introduced by segregation bias due to prezygotic isolation between the species or hybrid male over dominance in viability. We found that 15 of the 25 markers were not in the expected 1:2:1 (autosomes) or 1:1 (x-chromosome) ratios having excess heterozygotes, but there was no association between violation of these ratios and the location of the QTL (X^2^ = 0.45, p = 0.50). Thus, there is evidence of genetic variation for prezygotic isolation with a possible heterozygote advantage that may be worth mapping in future studies, but that variation did not introduce bias into the mapping for HMS. The linkage map, though, is likely only accurate for the population that was genotyped and cannot be applied to the F_2_-females that failed to mother hybrid sons, because two or more segregation distortion loci within a given marker interval can bias estimates of recombination rate [56]–[58].

### Architectural Complexity

The lack of a segregating main effect on the X-chromosome was surprising, as this chromosome has been generally shown to play a major role in the evolution of hybrid male sterility [14]. There may still be factors on the X-chromosome (or autosomes) segregating in the ancestral population as a whole whose effects were not captured by the two lines used in this study, and there also may be fixed factors on the X-chromosome that we cannot detect with this design. Additionally, there were several major discrepancies between the F_2_ and RF_2_ populations. Specifically, four main effect QTL were identified in the F_2_ population; two had largely additive effects on the 3^rd^ and 5^th^ chromosomes and two had largely non-additive effects on the 2^nd^ chromosome. In the RF_2_ population, however, only a single, largely additive QTL was found on the 3^rd^ chromosome. The combined population data set shows one 2^nd^ chromosome QTL and the 3^rd^ chromosome QTL. These unexpected results motivated further analyses of the underlying architectural complexity of HMS.

There are several possible explanations for the discrepancy between F_2_ and RF_2_ populations. First, we may have failed to detect QTL due to simple sampling variance between the F_2_ and RF_2_ populations. Second, the different compositions of the X-chromosomes in the reciprocal populations could have lead to an X-by-autosome interaction. Finally, an interaction between the nature of the cross (*e.g.* cytoplasm) and the QTLs could account for (or underlie) the observed differences between reciprocal crosses. We address each of these below.

*Sampling Variance*: To test whether the inevitable force of sampling variance was driving the differences observed between the F_2_ and RF_2_ populations, we subsampled from the datasets to test for consistency of the signal. From each population 100 datasets were created by randomly selecting 80% of the data points from the original dataset. Complete mapping analyses, including permutations for significance thresholds, were then performed on the subsampled datasets. Despite the reduced power of the smaller datasets, the QTLs found on the full dataset remained detectable in between 27% and 70% of the subsampled datasets (Figure S2), while spurious QTL were only detected in a maximum of 24% (*e.g*. see left portion of chromosome 5 in the F_2_ dataset). Subsampling indicates that QTL identified in the complete dataset remain detectable in a large portion of the subsets despite variation in sampling and spurious QTL (those not detected in the complete dataset but in the subsets) rarely arise due to sampling variance. The consistency between our subsampled datasets and our complete dataset suggests that our findings are fairly robust to variation in sampling and that the differences between the F_2_ and RF_2_ are likely biologically real, though some of the differences could still be due in part to sampling variance.

*Inheritance of the X-chromosome*: If an important X-linked genetic difference between the parental lines interacts with the autosomes, the cross direction will affect the frequency of that factor in the mapping population. This, in turn, would influence the detection and nature of autosomal QTL that interact with the X. The X-linked factor could primarily influence the penetrance or expression of the genetic variation at the autosomal factor and thus have a significant epistatic effect without having a main effect. To test for X-by-autosome interactions, as well as for other epistasis, we looked for significant pairwise interactions between all markers across the combined dataset (n = 304). We found seven interactions with a FDR significance of 0.05 or less, two of which were between the X-chromosome and the autosomes (Table 3, Figure 2). Epistatic interactions between the X-chromosome and the autosomes therefore could be contributing to some of the differences observed between the F_2_ and RF_2_ populations. We also found 5 inter-autosomal epistatic interactions. Notice that markers on the 4^th^ chromosome were involved in five of the seven interactions, thus the 4^th^ chromosome appears to be playing an important epistatic role in the HMS in this system worthy of additional study. Only one of nine markers involved in these pairwise interactions (m4_9_1 on the 5^th^ chromosome) resides in or near a main effect QTL. Two additional markers (a1_2_1 on chromosome 3 and m1_10_11 on chromosome 4) did not appear in the list of significant marker by marker interactions, but exhibited significant deviations from the additive expectation after a Bonferroni correction, indicating that there are at least two other unidentified epistatic interactions. Taken together, not only does the X-chromosome appear to play an epistatic role in shaping the genetic architecture of HMS in this system, but so does within-species epistasis throughout the rest of the genome.

**Table 3:** Significant Marker-Marker Interactions

Marker A	Marker B	Chromosome A	Chromosome B	p-value	FDR
*dmojx030*	*a3_11_1*	*X*	*2*	*0.00112*	*0.0496*
*m2_18_2*	*m2_19_2*	*X*	*4*	*0.00072*	*0.0496*
*a3_11_1*	*m2_19_2*	*2*	*4*	*0.0006*	*0.0496*
*m2_17_15*	*m2_19_2*	*3*	*4*	*0.00116*	*0.0496*
*dmoj4050*	*dmoj4060*	*4*	*4*	*0.00116*	*0.0496*
**m2_19_2**	**m4_9_1**	**4**	**5**	**0.00013**	**0.0393**
*a3_12_6*	*m4_9_1*	*5*	*5*	*0.0003*	*0.0453*

Markers and their corresponding chromosomes with interaction effects with a False Discovery Rate (FDR) of 0.05 or less. Interactions significant after a Bonferroni correction are in bold and interactions significant at an FDR of 0.05 are italicized.

**Figure 3:**
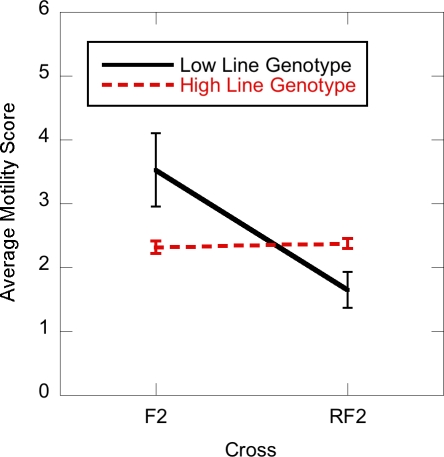
Interaction effect between marker genotype and cross-type on motility score. The average motility score of the homozygous marker genotype on the second chromosome (dmoj2210-High or Low parental line) relative to which cross it was derived (F_2_ or RF_2_). There is a significant interaction between marker and cross type.

*Maternal effects:* Cytoplasmic factors may also underlie interactions between the direction of the cross and the QTLs. We tested for marker by cross interactions for the four markers showing main effects and found a significant interaction effect of marker dmoj2210 on the 2nd chromosome with cross (p = 0.0142), after controlling for the main effect of the 3^rd^ chromosome (Figure 3). This marker did not show evidence of interacting with the X-chromosome in the test for epistasis above. Chromosome 2 is where the large and opposing non-additive QTL were found in the F_2_ population but not in the RF_2_ populations. The significant cross by marker interaction on the 2^nd^ chromosome is a likely contributor to the differences seen in the mapping for the two crosses. The cross effect is not necessarily independent of the epistatic effect of the X –chromosome found above. But since dmoj2210 did not show any evidence of interacting with the X-chromosome, it is likely that the cytoplasm plays a role in HMS. Thus, both the X-chromosome and the cytoplasmic environment should be further explored in future studies of HMS.

## Discussion

The first question we proposed to answer in this study was: what is the basic architecture of HMS in the early stages of its evolution? Is HMS caused by only a few factors of large effect or does it have a more complex basis, involving multiple loci and epistatic interactions? An earlier study [33] showed evidence of multiple polymorphic factors for HMS within *D. mojavensis* and additional data show genetic polymorphisms for HMS segregating in the sister species *D. arizonae* (Reed, *unpublished*). Our present QTL study reveals that HMS, even at the earliest stages of speciation, can be surprisingly complex. We found within-population variation not only for several QTL of main effect, but also for significant within-species epistatic interactions. Similar complexity has been seen in other systems, such as with the *Lhr-Hmr* gene pair, where the incompatibility requires an epistatic interaction with the hybrid genetic background to be expressed [59]. There is likely to be more segregating genetic variation in this species that was not captured by these two particular mapping lines, and fixed genetic factors that could not be detected by this design; potentially both of which further contribute to the complexity of this trait. While previous investigations on more diverged species pairs [10] have revealed a multi-factored genetic basis for post-zygotic isolation, these studies were unable to determine whether the polygenicity had arisen early or was a byproduct of long divergence time. We show that HMS has a polygenic and epistatic basis even early in the evolutionary process, before the ability to produce HMS is fixed within a species. These findings are consistent with the complex genetic basis of early postzygotic isolation found in *Tribolium* [28], [29]

In addition to the unexpected complexity of HMS in this incipient species pair, we were surprised to discover no main-effect QTL segregating on the X-chromosome, though we did find an epistatic effect. While we may have lacked the statistical power to detect X-linked main effects, main effects were detected on other chromosomes indicating that sample size alone is not the full explanation. There may still be fixed X-linked effects that could not be detected by this design, and fixed effects on the X are more likely due its smaller effective population size. Demuth and Wade did demonstrate the role of epistatic variation on the X-chromosome on Haldane's Rule both theoretically [13] and empirically [28], [29]. We also found such a role for the X-chromosome in our epistasis tests.

Finally, our data show a striking difference between reciprocal crosses, suggesting a potential role for maternal effects and genome-wide epistatic variation on HMS. Cytoplasmic and epistatic effects have been demonstrated in other systems as well [24], [28], [29], [59]. Zouros and colleagues have mapped the genetic basis of HMS resulting from the reciprocal cross (female *D. arizonae* mated to male *D. mojavensis*) to the third, fourth and Y-chromosomes [40], [41], [60]–[62]. Whether or not the third chromosome effect in the cross using *D. arizonae* mothers is a function of the same factor or factors as revealed in our study remains unknown.

The second question we proposed to answer was: what can the genetic architecture of HMS tell us, in conjunction with other information, about its evolution? Substantial within-species natural polymorphism for between species postzygotic isolation has been characterized in many independent taxa thus far, including at least nine *Drosophila* species groups, [33], [34], [43], [63]–[70], *Tribolium* beetles [71], [72], *Chorthippus parallelus* grasshoppers [73], *Mus musculus* [74], *Xiphophorus* fish [75], *Crepis* hawksberg weed [76], *Gossypium* cotton [77] and *Mimulus* monkey flowers [78]. Thus, intraspecific polymorphism for postzygotic isolation appears to be a common phenomenon. Shuker *et al*. [73] argue that witnessing substantial within-species polymorphism for hybrid incompatibilities means that loci contributing to the hybrid phenotype are likely to be neutral or nearly-neutral in the parental species' genetic background. Finding that a trait is polymorphic at multiple loci, supports the role of drift and/or balancing selection in the genetics of the trait. If directional selection was occurring on the genes related to the trait, no matter what the within- species function of the genes, they would be polymorphic for only the briefest of times [79]. The probability of catching multiple polymorphic loci under directional selection would be minute. Considering that there is evidence of balancing selection on male reproductive proteins in several systems [80]–[83] it is not unreasonable to hypothesize that it might also be playing a role in HMS.

A handful of genes causing hybrid incompatibilities have been identified in other species and all but one (a gene transposition [84]) show evidence of being under positive selection (*OdsH* in *D. mauritiana*, [85]; *Xmrk-2* in *Xiphophorus*, [86]; *Nup96*, *Hmr*, *Lhr* in the *D. melanogaster/D. simulans* species pair [24], [87], [88]), leading to the impression that most postzygotic speciation genes experience directional selection. Given our findings with *D. mojavensis* and in other systems discussed above, however, we believe it is premature to discount the role of drift and balancing selection in speciation. Empirically, we need to characterize more completely the genetic architecture of incompatibility phenotypes during early stages to find reproductive isolation genes before they are fixed and test those loci for evidence of selection. In the present study we have begun the empirical aspect of this process.

## Supporting Information

Table S1 Primers for Microsatellites.
(0.06 MB DOC)

Figure S1 Linkage map for D. mojavensis. Distance between markers given in centiMorgans.
(0.30 MB DOC)

Figure S2 Subsampling effect on QTL detection. Bar graphs of count (right x-axis, out of 100) of 80%-subsampled datasets at each mapping interval showing a likelihood ratio greater than the significance threshold determined by permutation for each subset. Solid lines indicate likelihood ratio (left x-axis) calculated from the complete dataset. A. F2-sons dataset. B. RF2-sons dataset. Subsampling indicates that QTL identified in the complete dataset remain detectable in a large portion of the subsets and spurious QTL (those not detected in the completely dataset) rarely arise due to sampling variance. Thus, patterns observed in the complete dataset appear robust to sampling variance.
(0.16 MB DOC)
