# Supplementary material for: Genetic Architecture of Hybrid Male Sterility in Drosophila: Analysis of Intraspecies Variation for Interspecies Isolation
Source: PLoS One. 2008 Aug 27;3(8):e3076. doi: 10.1371/journal.pone.0003076 (PMC2517651; doi:10.1371/journal.pone.0003076)
Supplement: Table S1 — Primers for Microsatellites. (0.06 MB DOC) [file pone.0003076.s001.doc]

Supplemental Table 1: Primers for Microsatellites.

| **Marker** | **Chromosome** | **Forward Primer** | **Reverse Primer** |
| --- | --- | --- | --- |
| M2_18_2 | X | GATGACATCAGCGAGACTTCC | CCAAATTGCACATCAACAGC |
| A3_8_9 | 2 | GCAACAGCAGCAACAGAAGC | ACTAGCCCACACACCCTACC |
| A3_11_1 | 2 | CAACAACAACTGCAGCAACC | TGTCATTCGGCAGTGTATCC |
| A3_10_13 | 3 | CGTTTTTCGCGTTGTCATC | TTTCGGAATGCTCCTATTCG |
| A3_16_5 | 3 | AATGCAATAAAGCGCGAATC | TGAAGAGTTGCAATTGAAGCAG |
| chrom3dup | 3 | GAAGCCCACTGGCAAACG | ACAGGGCAGACTTCATTAGC |
| M2_17_15 | 3 | GCATATCAATGTCCTGTTATCAAGC | ATTTCCCCATCTGACTGTCG |
| A1_2_1 | 3 | CGAAATTCAGCACCAAAGC | AGCCCCAGCTACATGTTCG |
| A2_13_1 | 4 | CAGAAATCGTTTCATTCATGC | CGCTTGGACAACTTTCAGC |
| M2_19_2 | 4 | CCTTATCGCTGCTCGACTCC | AGGAAAACTTCAGCCAGACG |
| chrom4dup | 4 | GCATTCATATGCATTTAATTTTCG | AGACTCCAACGCGATTTTCC |
| M1_10_11 | 4 | TTGTCAGCATTTGATGAGC | TTTGTGCCAGCAATTATGTAGC |
| A2_10_3 | 5 | CCAATGCTGTTGCTCTTACG | CGCTCGCTATTATCCTCTCC |
| M4_9_6 | 5 | TCAACTGGAAGCTGTTAAATATCG | CATGCATCAGGCTTATCTCC |
| M4_9_1 | 5 | CCTGCAACTCAAACTTCACC | GCTTCAGCTACCCAAAAGTCC |
| A3_12_6 | 5 | ACGCATTTGCTGTGATTCG | GAAAATGTCAAGGGGACAGC |
