# Supplementary material for: Genetic Architecture of Hybrid Male Sterility in Drosophila: Analysis of Intraspecies Variation for Interspecies Isolation
Source: PLoS One. 2008 Aug 27;3(8):e3076. doi: 10.1371/journal.pone.0003076 (PMC2517651; doi:10.1371/journal.pone.0003076)

Supplemental Figure 1: Linkage map for *D. mojavensis*. Distance between markers given in centiMorgans.


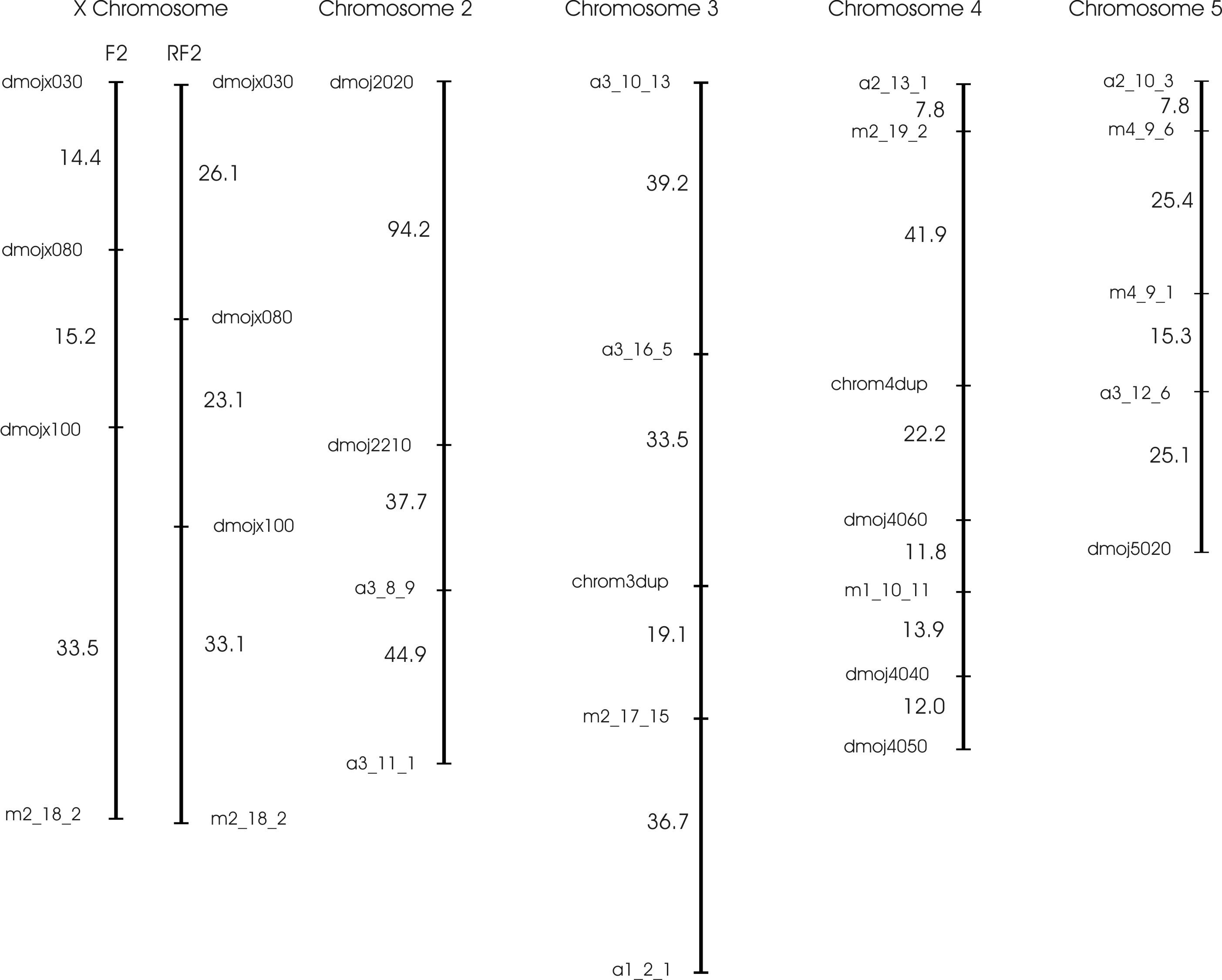

Supplement: Figure S1 — Linkage map for D. mojavensis. Distance between markers given in centiMorgans. (0.30 MB DOC) [file pone.0003076.s002.doc]
