# Supplementary material for: Genetic Architecture of Hybrid Male Sterility in Drosophila: Analysis of Intraspecies Variation for Interspecies Isolation
Source: PLoS One. 2008 Aug 27;3(8):e3076. doi: 10.1371/journal.pone.0003076 (PMC2517651; doi:10.1371/journal.pone.0003076)

Supplemental Figure 2: Subsampling effect on QTL detection. Bar graphs of count (right x-axis, out of 100) of 80%-subsampled datasets at each mapping interval showing a likelihood ratio greater than the significance threshold determined by permutation for each subset. Solid lines indicate likelihood ratio (left x-axis) calculated from the complete dataset. A. F2-sons dataset. B. RF2-sons dataset. Subsampling indicates that QTL identified in the complete dataset remain detectable in a large portion of the subsets and spurious QTL (those not detected in the completely dataset) rarely arise due to sampling variance. Thus, patterns observed in the complete dataset appear robust to sampling variance.


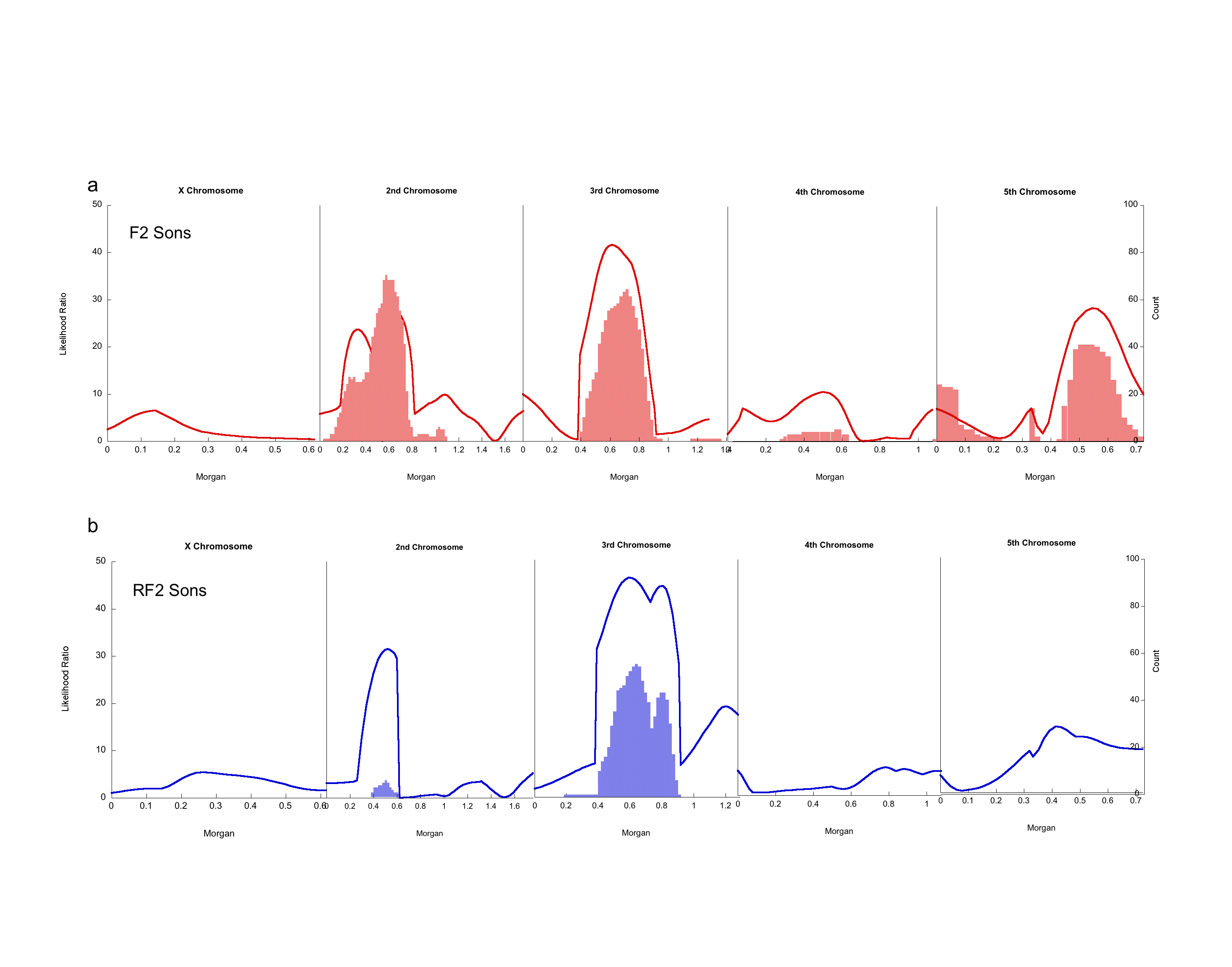

Supplement: Figure S2 — Subsampling effect on QTL detection. Bar graphs of count (right x-axis, out of 100) of 80%-subsampled datasets at each mapping interval showing a likelihood ratio greater than the significance threshold determined by permutation for each subset. Solid lines indicate likelihood ratio (left x-axis) calculated from the complete dataset. A. F2-sons dataset. B. RF2-sons dataset. Subsampling indicates that QTL identified in the complete dataset remain detectable in a large portion of the subsets and spurious QTL (those not detected in the completely dataset) rarely arise due to sampling variance. Thus, patterns observed in the complete dataset appear robust to sampling variance. (0.16 MB DOC) [file pone.0003076.s003.doc]
